# Random amplified polymorphic DNA-based molecular heterogeneity analysis of *Salmonella enterica* isolates from foods of animal origin

**DOI:** 10.14202/vetworld.2019.146-154

**Published:** 2019-01-26

**Authors:** Surendra Singh Shekhawat, Abhishek Gaurav, Bincy Joseph, Hitesh Kumar, Nirmal Kumar

**Affiliations:** 1 Department of Veterinary Public Health and Epidemiology, College of Veterinary and Animal Science, Navania, Vallabhnagar, Udaipur, Rajasthan, India; 2 Department of Veterinary Microbiology, College of Veterinary and Animal Science, Navania, Vallabhnagar, Udaipur, Rajasthan, India

**Keywords:** *Salmonella*, random amplified polymorphic DNA, foods of animal origin, phylogram

## Abstract

**Aim:**

This study aims to study the significance of random amplified polymorphic DNA (RAPD) typing in heterogeneity analysis of *Salmonella* serovars, isolated from foods of animal origin.

**Materials and Methods:**

*Salmonella* serovars isolated and identified from different foods of animal origin such as meat, milk, and egg by standard bacteriological methods. DNA isolated from all 10 isolates which are confirmed by biochemical and serotyping methods and then RAPD was performed using the primers OPB 10, primer 1290, NSC I, NSC II, and primer 3. Then, RAPD data were analyzed using the BioNumerics software, Belgium, Germany.

**Results:**

RAPD polymerase chain reaction (PCR) using five primers, namely OPB 10, primer 1290, NSC I, NSC II, and primer 3, classified the 10 isolates into 9, 10, 10, 7, and 10 RAPD-PCR types with discriminating powers of 0.1987, 0.423, 0.50889, 0.1842, and 0.2582, respectively. The phylogram constructed with NSC I profile classified isolates based on geographical origin. Primer 1290, NSC II, and primer 3 produced some uniform bands in all isolates indicating their binding ability in conserved genomic region. This study revealed that RAPD profile can be best used for finding out the heterogeneity at molecular level of *Salmonella* isolates in combination with other molecular and phenotypic typing techniques. Thus, our results support earlier observation of its significance by different workers on different *Salmonella* serotypes.

**Conclusion:**

Repeatability of RAPD-PCR is insufficient to distinguish genetic differences among *Salmonella* serovars.

## Introduction

Foodborne diseases are a serious a public health concern in food industry and *Salmonella* organisms are most frequently isolated bacterial agents of foodborne outbreaks [1]. Gram-negative *Salmonella* is a major health problem worldwide that causes typhoidal and non-typhoidal salmonellosis. Typhoidal and non-typhoidal illnesses cause millions of cases yearly with significant economic losses and even human deaths [2]. Most cases of non-typhoidal *Salmonella* (NTS) disease are associated with consumption of contaminated foods of the animal origin, particularly poultry meat and in some instances vegetables [3,4]. *Salmonella* serovars are responsible for infections occurring in developing as well as developed world and have been a major concern in terms of economic burden due to high morbidity [5]. *Salmonella* is an important cause of foodborne (alimentary) health problems in humans [6]. *Salmonella* was the second laboratory confirmed etiological agent accounting for 229 (30%) reported food poisoning outbreaks in the United States [7].

The development of polymerase chain reaction (PCR) technology has allowed the specific amplification of particular target segments of DNA. Several PCR-based assays have been developed for rapid detection of *Salmonella* spp. [8]. Serotyping is most widely used phenotypic method, but it fails to provide appropriate information due to complex serotyping scheme and lack of comparison among different laboratories, thereby limiting its application to the reference laboratories only [9]. Genotypic characterization of bacteria which are foodborne like *Salmonella* is very important to determine the genetic diversity of strains. *Salmonella* is a diverse group of bacteria with a large number of serotypes and strains present in various hosts which include animal, birds, and humans. Moreover, due to diverse ecological habitats of this bacterium, there is a need of comparing the strains from different sources to determine the clonal variation or similarity for physiological studies. Genotypic characterization helps to understand the complete epidemiology of the disease and aids in understanding the evolutionary pathways of various strains originating from different ecological niches. Therefore, various novel genotyping methods such as variable number tandem repeats (VNTR), multilocus VNTR, multilocus sequence typing, and ERIC-PCR have been developed and utilized [10-13] to delineate epidemiological relationships among various isolates even within the same phage types [14]. However, the search continues for the easy to use efficient method capable of differentiating strains of similar phenotype [15]. The application of random amplified polymorphic DNA (RAPD) analysis based on random amplification of genomic DNA fragments through short arbitrarily designed primers is an attractive alternative and has the potential to detect polymorphism throughout the entire genome as compared to other techniques [16].

In the present study, we tried to analyze the significance of RAPD typing in heterogeneity analysis of *Salmonella* serovars, isolated from foods of animal origin.

## Materials and Methods

### Ethical approval

In the present investigation, we have not used any live animals, therefore; no ethical approval was needed for the present study.

### Bacteria

A total of 10 *Salmonella* isolates obtained from foods of animal origin [17] were used in the study. All the 10 positive *Salmonella* isolates were sent to Central Research Institute (CRI), Kasauli, Himachal Pradesh, for further serotyping.

### DNA isolation

DNA isolated from all the 10 biochemically confirmed isolates using HiMedia TM Bacterial Genomic DNA Purification Kit following the manufacturer’s instructions supplied along with the kit with suitable modifications. Briefly, 1.5 ml of overnight broth culture was pelleted by centrifugation at 15,000 rpm for 2 min. The supernatant was discarded, and pellet was resuspended in 180 µl of lysis solution AL. After adding 200 µl lysis solutions C_1_, it was vortexed for 15 s and then incubated at 55°C for 10 min. Then, 200 µl of ethanol (95-100%) was added to the lysate and mixed thoroughly by vortexing for 15 s. The lysate so obtained was transferred into spin column and centrifuged at 10,000 rpm for 1 min. The flow-through liquid was discarded and placed in a new 2 ml collection tube. Then, 500 µl of prewash solution was added to the spin column and centrifuged at 10,000 rpm for 1 min. The flow-through was again discarded, and same collection tube was used. A volume of 500 µl of diluted wash solution was added to column and centrifuged at 15,000 rpm for 3 min and spin again at same speed for the additional 1 min to dry the column. The HiElute spin column was placed on a fresh tube and 100 µl of elution buffer which was kept in a water bath at 65°C for 30 min. The column was incubated at room temperature for 5 min followed by centrifugation at 10,000 rpm for 1 min. The spin column was then removed, and the collected DNA was stored at −20°C for further use. The concentration of DNA isolated was estimated spectrophotometrically using Biospectrometer (Eppendorf, USA) using the following formula: DNA concentration (µg/µl) = [OD_260_ × dilution factor × 50 mg/ml]/1000. The purity was checked as ratio of OD_260_ and OD_280,_ and the integrity of the purified DNA was assessed by running it in 0.7% agarose gel.

### RAPD-PCR

RAPD-PCR was performed using genomic DNA of *Salmonella* isolates as a template with the random primers (Xcelris) mentioned in Table-1 [18-20]. Each PCR mixture consisted of 1 µg of template DNA, 2 pM solution of each primer (Xcelris, India), 5 µl 10× PCR buffer (Thermo Scientific, USA), 3 mM MgCl_2_ (Thermo Scientific, USA), 300 µM each nucleotides (Thermo Scientific, USA), and 3.75 units of *Taq* DNA polymerase (Thermo Scientific, USA) in 50 µl PCR reaction mix. PCR program for NSC I primer included initial denaturation at 94°C for 5 min, followed by 40 cycles of denaturation (94°C for 1 min), annealing (25°C for 45 s), and extension (72°C for 1 min). Final extension was carried out at 72°C for 7 min. For primers NSC II, 1290, OPB-10, and primer 5, PCR reaction mix and cycling conditions were same with the exception of their annealing temperature which was adjusted at 29°C, 27°C, 30°C, and 27°C respectively. PCR products were characterized by submarine gel electrophoresis on 0.9% agarose gel. After electrophoresis, the gel was visualized in gel documentation system, and picture was taken. 100 bp DNA ladder (Thermo Scientific, USA) and 1 kb DNA ladder (Thermo Scientific, USA) were used as molecular weight markers.

**Table-1:** Sequences of different primers used for RAPD analysis of *Salmonella enterica* isolates.

Target genes	Primer sequence (5’→3’)	Melting temperature (°C)	Type	References
OPB-10	5’- CGT CTG GGA C-3’	34	RAPD	[18]
Primer 1290	5’- GTG GAT GCG A-3’	32	RAPD	[19]
NSC I	5’- AGG ACC AGG-3’	30	RAPD	[19]
NSC II	5’- AGG GCC CGG G-3’	34	RAPD	[19]
Primer 3	5’- CGT GCA CGC-3’	32	RAPD	[20]

### Analysis of RAPD data

The banding patterns from RAPD were analyzed with BioNumerics Software version 7.5 (Applied Maths, Ghent, Belgium). The gel images of respective genotypic technique were uploaded as tiff format. The gel image of the genotyping PCR was processed in four steps, namely strip creation, calculation of densitometric curves, normalization of gel image, and detection of bands on the gel. The strip creation step identifies individual lane of the sample and defines the area of it to cut from the main gel. In the densitometric step, the software builds the peaks on the basis of intensity of bands and detects the strength of the positivity of each band. The normalization step helps to save the changes in the edited image and helps to set a reference marker. Finally, in the band calculation step, each of the bands identified on the gel is compared against the reference marker and compared with other bands present on the gel. After processing the gel image, each of the lane data was added to the database of BioNumerics Software version 7.5, in particular, genotyping PCR technique. The data were used to make a comparison of the lanes for the creation of dendrogram by similarity matrix using the Dice coefficient with optimization of 1%. The dendrograms were obtained by means of Unweighted Pair Group Method with Arithmetic Average clustering algorithm.

The numerical index of discrimination (D) of each primer was also calculated using the Simpson’s index of diversity [21] using the following formula:


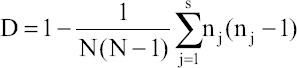


Where N is the total number of strains in the sample population, s is the total number of types described, and n is the number of strains belonging to j^th^ type.

## Results and Discussion

Nowadays, advances in molecular biological tools utilizing various typing techniques make it simpler to differentiate isolates of *Salmonella* serovars. Keeping this in view, the attempts were made to evaluate the molecular heterogeneity among *Salmonella* isolates from foods of animal origin using RAPD-PCR. *Salmonella enterica* isolates obtained from foods of animal origin were subjected to serotyping at CRI, Kasauli, Himachal Pradesh, India. The results of serotyping are presented in Table-2.

**Table-2:** Details of the serotype of *Salmonella enterica* isolates.

Isolate No	Serotype	Source
S98	*Salmonella* Lindenburg	Chicken
S115	*Salmonella* Lindenburg	Chicken
S158	*Salmonella* Lindenburg	Chicken
S221	*Salmonella* Rough	Chevon
S306	*Salmonella* Rough	Egg
S410	*Salmonella* Enteritidis	Egg
S453	*Salmonella* Lindenburg	Chicken
S485	*Salmonella* Rough	Milk
S522	*Salmonella* Rough	Milk
S570	*Salmonella* Typhimurium	Milk

The application of RAPD analysis [22-24] based on random amplification of genomic DNA fragments through short arbitrarily designed primers allows one to start a blind walk through whole genomic DNA of an organism. The discriminatory power of this typing method can be enhanced by the use of more than one primer. For this reason, this study incorporated five random primers for differentiating isolates of *Salmonella* species. It was observed that all five primers employed were capable of elucidating polymorphic amplification patterns in all isolates.

**Figure-1:**
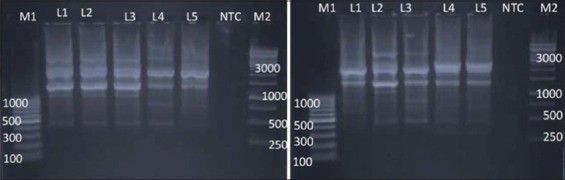
Random amplified polymorphic DNA- polymerase chain reaction profiles of *Salmonella* *enterica* isolates with OPB 10. Plate 1- Lane M1: 100 bp ladder; Lane 1: *Salmonella* Lindenburg; Lane 2: *Salmonella* Lindenburg; Lane 3: *Salmonella* Lindenburg; Lane 4: *Salmonella* Rough; Lane 5: *Salmonella* Rough; Lane 6: NTC; Lane M2:1 kb ladder and Plate 2 – Lane M1: 100bp ladder; Lane 1: *Salmonella* Enteritidis; Lane 2: *Salmonella* Lindenburg; Lane 3: *Salmonella* Rough; Lane 4: *Salmonella* Rough; Lane 5: *Salmonella* Typhimurium; Lane 6: NTC; Lane M2: 1 kb ladder.

**Figure-2:**
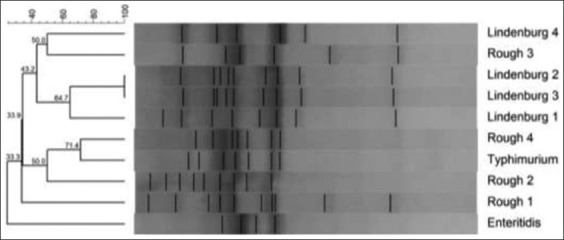
Dendrogram of random amplified polymorphic DNA - polymerase chain reaction profile of *Salmonella* *enterica* isolates with OPB 10.

RAPD-PCR using primer OPB-10 was successfully able to produce fingerprints of all the 10 isolates of *Salmonella* spp. tested (Figure-1). Some bands are common in all the isolates indicating the ability of this primer to bind to some conserved regions of *Salmonella* genomic DNA. On the basis of dendrogram prepared using RAPD-PCR assay, 10 isolates of *Salmonella* could be divided into nine RAPD-PCR types (Figure-2). A maximum number of bands (10) produced by *Salmonella* Rough-1 and minimum number of bands (4) produced by *Salmonella* Enteritidis isolates. The analysis of fingerprints in the dendrogram displayed that four isolates of *Salmonella* Lindenburg showed three different types of fingerprints, hence, dividing them into three clonal groups. This indicates the ability of OPB-10 primer to discriminate within the serovar Lindenburg also.

Similarly, Sumithra *et al*. [25] reported the high discriminative power OPB 10 primer to discriminate between the isolates of *Salmonella* Typhimurium. We observed an average genetic similarity between the four isolates of *Salmonella* Lindenburg (69.3%). The four *Salmonella* Rough isolates were separated into four different RAPD types with average genetic similarity of 33.9%. The genetic similarity between serotype *Salmonella* Lindenburg and *Salmonella* Enteritidis was 33.3% (66.7% average genetic diversity). The genetic similarity between serotype *Salmonella* Lindenburg and *Salmonella* Typhimurium was 33.9% (66.1% average genetic diversity). The genetic similarity between serotype *Salmonella* Enteritidis and *Salmonella* Typhimurium was 33.3% (66.7% average genetic diversity). The *Salmonella* isolates from chicken showed an average similarity of 69.3% (30.7% average genetic diversity). Genetic similarity between *Salmonella* spp. isolated from milk ranged 33.9-71.4% with an average similarity of 52.65% (47.35% average genetic diversity). Average genetic similarity between *Salmonella* spp. isolated from egg was 33.3% (66.7% average genetic diversity). Average similarity between *Salmonella* spp. isolated from egg and chicken is 33.3% (66.7% average genetic diversity). The discrimination power of OPB 10 was calculated as 0.1987. Our results were contrary to the reports of Sumithra [26] who reported that OPB 10 primer has a better discrimination power (0.88312) to discriminate between different isolates of *Salmonella* Typhimurium. As per the results obtained in this study, the discriminative power of OPB-10 primer between the different serovars is very poor.

**Figure-3:**
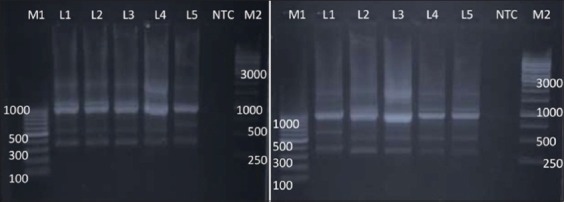
Random amplified polymorphic DNA - polymerase chain reaction profiles of *Salmonella* *enterica* isolates with primer 1290. Plate 1 Lane M1: 100 bp ladder; Lane 1: *Salmonella* Lindenburg; Lane 2: *Salmonella* Lindenburg; Lane 3: *Salmonella* Lindenburg; Lane 4: *Salmonella* Rough; Lane 5: *Salmonella* Rough; Lane 6: NTC; Lane M2: 1kb ladder and Plate 2-5: *Salmonella* Typhimurium; Lane 6: NTC: Lane M2: 1 kb ladder.

**Figure-4:**
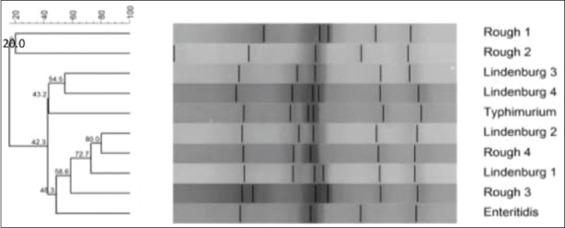
Dendrogram of random amplified polymorphic DNA - polymerase chain reaction profile of *Salmonella* *enterica isolates* with primer 1290.

RAPD-PCR using primer 1290 was successfully able to produce fingerprints of all the 10 isolates of *Salmonella* spp. tested (Figure-3). Here also, most of the bands are commonly indicating the ability of this primer to bind to the conserved regions of *Salmonella*. On the basis of dendrogram prepared using RAPD-PCR assay, 10 isolates of *Salmonella* could be divided into 10 RAPD-PCR types (Figure-4). The analysis of fingerprints in the dendrogram of four isolates of *Salmonella* Lindenburg showed four different types of fingerprints, hence, dividing them into four clonal groups. We observed an average genetic similarity between four isolates of *Salmonella* Lindenburg to be 56.5% (43.5% average genetic diversity). The four *Salmonella* rough strains were separated into four different RAPD types with an average genetic similarity of 39.8% (60.2% average genetic diversity). The genetic similarity between serotype *Salmonella* Lindenburg and *Salmonella* Enteritidis was 50.9% (66.7% average genetic diversity). The level of similarity ranged between 43.2% and 58.6%. The genetic similarity between serotype *Salmonella* Lindenburg and *Salmonella* Typhimurium was 33.9% (66.1% average genetic diversity). The level of similarity ranged between 43.2 and 48.3. The genetic similarity between serotype *Salmonella* Enteritidis and *Salmonella* Typhimurium was 42.3% (57.7% average genetic diversity).

The genetic similarity among *Salmonella* spp. from chicken ranged between 42.3% and 72.7% with an average similarity of 56.5% (43.5% average genetic diversity). Average similarity between *Salmonella* spp. isolated milk ranged from 43.2 to 58.6%. Average genetic similarity between *Salmonella* spp. isolated from egg was 21% (79% average genetic diversity) and the similarity between isolates from egg and chicken was 34.65%. The Simpson diversity index of primer 1290 was 0.423.

**Figure-5:**
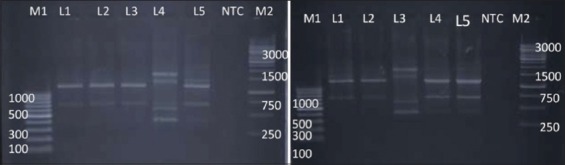
Random amplified polymorphic DNA - polymerase chain reaction profiles of *Salmonella* *enterica* isolates with NSC I. Plate 1-Lane M1: 100 bp ladder; Lane 1: *Salmonella* Lindenburg; Lane 2: *Salmonella* Lindenburg; Lane 3: *Salmonella* Lindenburg; Lane 4: *Salmonella* Rough; Lane 5: *Salmonella* Rough; Lane 6: NTC; Lane M2: 1 kb ladder and Plate 2 – Lane M1: 100 bp ladder; Lane 1: *Salmonella* Enteritidis; Lane 2: *Salmonella* Lindenburg; Lane 3: *Salmonella* Rough; Lane 4: *Salmonella* Rough; Lane 5: *Salmonella* Typhimurium; Lane 6: NTC; Lane M2: 1 kb ladder.

**Figure-6:**
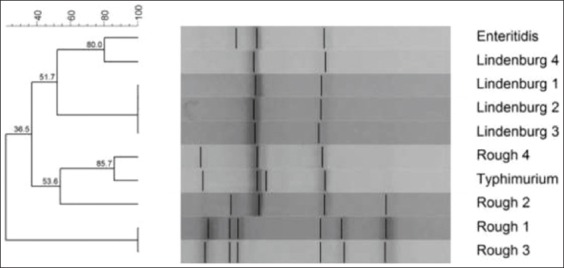
Dendrogram of random amplified polymorphic DNA - polymerase chain reaction profile of *Salmonella* *enterica isolates* with NSCI.

RAPD-PCR using NSC I also produce fingerprints of all the 10 isolates of *Salmonella* spp. tested (Figure-5). On the basis of dendrogram prepared, 10 isolates of *Salmonella* could be divided into seven RAPD-PCR types (Figure-6). The analysis of fingerprints in the dendrogram of four isolates of *Salmonella* Lindenburg showed two different types of fingerprints, hence, dividing them into two clonal groups. We observed an average genetic similarity between four isolates of *Salmonella* Lindenburg, 75.85% (24.15% average genetic diversity). The level of similarity ranged between 51.7% and 100%. The four *Salmonella* Rough strains were separated into three different RAPD types with average genetic similarity of 68.25% (31.75% average genetic diversity). The genetic similarity between serotype *Salmonella* Lindenburg and *Salmonella* Enteritidis was 65.85% (34.15% average genetic diversity). The level of similarity ranged between 51.7% and 80%. The genetic similarity between serotype *Salmonella* Lindenburg and *Salmonella* Typhimurium was 51.7% (48.3% average genetic diversity) and between serotype *Salmonella* Enteritidis and *Salmonella* Typhimurium was 36.5% (63.5% average genetic diversity). The discrimination power of NSC I was 0.50889.

The genetic similarity among *Salmonella* spp. from chicken showed a genetic similarity ranged between 51.7% and 100% with an average similarity of 75.85% (24.15% average genetic diversity). Average similarity between *Salmonella* spp. isolated from milk was 62% (38 % average genetic diversity) and the similarity between the isolates from egg was 36.5% (63.5% average genetic diversity). Average similarity between *Salmonella* spp. isolated from egg and chicken is 58.25% (41.75% average genetic diversity). This study point toward the power of NSC I primer to some extent group the *Salmonella* isolates based on their host origin. Sumithra *et al*. [25] also reported the ability of NSC I primer to cluster *Salmonella* Typhimurium isolates based on geographical origin. The potential ability of NSC I primer to discriminate *Salmonella* Gallinarum based on their geographical origin was also reported by Habtamu *et al*. [19].

**Figure-7:**
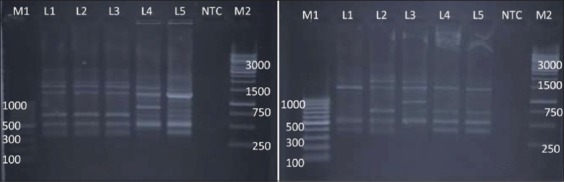
Random amplified polymorphic DNA - polymerase chain reaction profiles of *Salmonella* *enterica* isolates with NSC II. Plate 1- Lane M1: 100 bp ladder; Lane 1: *Salmonella* Lindenburg; Lane 2: *Salmonella* Lindenburg; Lane 3: *Salmonella* Lindenburg; Lane 4: *Salmonella* Rough; Lane 5: *Salmonella* Rough; Lane 6: NTC; Lane M2: 1 kb ladder and Plate 2 – Lane M1: 100 bp ladder; Lane 1: *Salmonella* Enteritidis Lane 2: *Salmonella* Lindenburg; Lane 3: *Salmonella* Rough; Lane 4: *Salmonella* Rough; Lane 5: *Salmonella* Typhimurium; Lane 6: NTC; Lane M2: 1 kb ladder.

**Figure-8:**
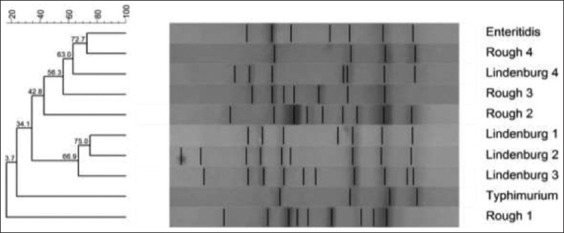
Dendrogram of random amplified polymorphic DNA - polymerase chain reaction profile of *Salmonella* *enterica* isolates with NSC II.

RAPD-PCR using NSC II was also able to produce fingerprints of all the 10 isolates of *Salmonella* spp. tested (Figure-7). On the basis of dendrogram prepared using RAPD-PCR assay, 10 isolates of *Salmonella* could be divided into 10 RAPD-PCR types (Figure-8). The analysis of fingerprints in the dendrogram of four isolates of *Salmonella* Lindenburg showed four different types of fingerprints, hence, dividing them into four clonal groups. We observed an average genetic similarity between four isolates of *Salmonella* Lindenburg, 61.56% (38.44% average genetic diversity). The four *Salmonella* Rough strains were separated into four different RAPD types with average genetic similarity of 33.25% (66.75% average genetic diversity). The level of similarity ranged between 23.7% and 42.8%. The genetic similarity between serotype *Salmonella* Lindenburg and *Salmonella* Enteritidis was 48.55% (51.45% average genetic diversity). The level of similarity ranged between 34.1% and 63%. The genetic similarity between serotype *Salmonella* Lindenburg and *Salmonella* Typhimurium was 34.1% (65.9% average genetic diversity). The genetic similarity between serotype *Salmonella* Enteritidis and *Salmonella* Typhimurium was 34.1% (65.9% average genetic diversity).

The genetic similarity among *Salmonella* spp. from chicken ranged between 42.8% and 75%. The average similarity was 61.56% (38.44% average genetic diversity) and for those isolates from milk ranged from 34.1% to 56.3% with an average similarity of 45.2% (54.8% average genetic diversity). Average genetic similarity between *Salmonella* spp. isolated from egg was 42.8% (57.2% average genetic diversity) and from egg and chicken was 48.55% (51.45% average genetic diversity). The discrimination power of NSC II was 0.1842. A report by Rezk *et al*. [27] showed maximum genetic variability among *Salmonella* Paratyphi B isolates using NSC II primer when compared to NSC I and NSC III.

Similarly, a study by Meenu [28] showed maximum genetic variability among *Salmonella* Gallinarum isolates of poultry origin using NSC II primer when compared to NSC I and 1290. Sumithra [26] also reported that D value of NSC II primer was better than 1290 similar to previous results, but it was similar to that of NSCI for the heterogeneity study of *Salmonella* Typhimurium. However, on the contrary to previous reports, this study showed that the discriminative power of NSC I primer is better than NSC II for differentiation of different *Salmonella* serovars. This variation may be due to the difference in reaction mixture such as the amount of magnesium chloride used in the reaction mixture. Also, difference in serotype and origin of the isolates may also be taken into consideration.

**Figure-9:**
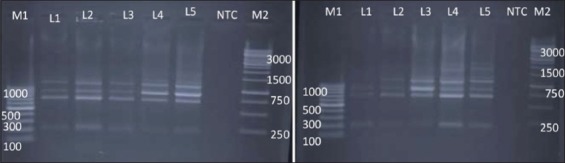
Random amplified polymorphic DNA - polymerase chain reaction profiles of *Salmonella* *enterica isolates* with primer 3. Plate 1 - Lane M1: 100 bp ladder; Lane 1: *Salmonella* Lindenburg; Lane 2: *Salmonella* Lindenburg; Lane 3: *Salmonella* Lindenburg; Lane 4: *Salmonella* Rough; Lane 5: *Salmonella* Rough; Lane 6: NTC; Lane M2: 1 kb ladder and Plate 2 – Lane M1: 100 bp ladder; Lane 1: *Salmonella* Enteritidis; Lane 2: *Salmonella* Lindenburg; Lane 3: *Salmonella* Rough; Lane 4: *Salmonella* Rough; Lane 5: *Salmonella* Typhimurium; Lane 6: NTC; Lane M2:1 kb ladder.

**Figure-10:**
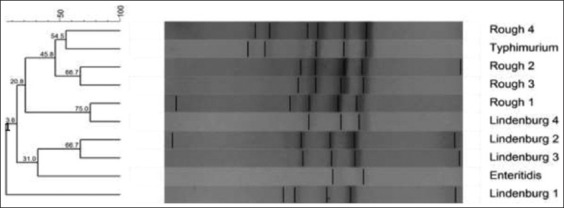
Dendrogram of random amplified polymorphic DNA - polymerase chain reaction profile of *Salmonella* *enterica* isolates with primer 3.

RAPD-PCR using primer 3 was also able to produce fingerprints of all the 10 isolates of *Salmonella* spp. tested (Figure-9). On the basis of dendrogram prepared using RAPD-PCR assay, 10 isolates of *Salmonella* could be divided into 10 RAPD-PCR types (Figure-10). The analysis of fingerprints in the dendrogram of four isolates of *Salmonella* Lindenburg showed four different types of fingerprints, hence, dividing them into four clonal groups. We observed an average genetic similarity between four isolates of *Salmonella* Lindenburg as 33.7% (66.3% average genetic diversity). The level of similarity ranged from 13.6% to 66.7%. The four *Salmonella* Rough strains were separated into four different RAPD types with average genetic similarity of 43.75% (56.25% average genetic diversity). The genetic similarity between serotype *Salmonella* Lindenburg and *Salmonella* Enteritidis was 21.8% (78.2% average genetic diversity) and between serotype *Salmonella* Lindenburg and *Salmonella* Typhimurium was 17.2% (82.8% average genetic diversity). The genetic similarity between serotype *Salmonella* Enteritidis and *Salmonella* Typhimurium was 13.6% (86.4% average genetic diversity).

*Salmonella* spp. from chicken showed a genetic similarity ranged between 13.6% and 66.7% with an average similarity of 33.7% (66.3% average genetic diversity) and those milk ranged from 45.8 to 54.8% with an average of 50.15% (49.85% average genetic diversity). Average genetic similarity between *Salmonella* spp. isolated from egg was 20.8% (79.2% average genetic diversity) and from egg and chicken is 25.9% (74.1% average genetic diversity). The discrimination power of primer 3 was 0.2582.

Due to polymorphism inherent in the sequence, as well as, the short length of primers used, the RAPD typing method resulted in a clustering of isolates into highly discriminating genetic trees [27]. Although relatively few samples were used in the study, the data suggest that RAPD typing is discriminatory; it is easy to interpret and constitute a low-cost method to type the various *Salmonella* serovars. These observations revealed that RAPD profile could be best used for finding out the heterogeneity at the molecular level of *Salmonella* isolates in combination with other molecular and phenotypic typing techniques. Thus, our results support the earlier observation of its significance by different workers on different *Salmonella* serotypes [28-35].

## Conclusion

The high discriminative power of NSCI revealed the power of this primer as a potential candidate in RAPD analysis of *Salmonella* isolates, and further, it can be used for the epidemiological studies for the classification of *Salmonella* isolates based on their geographical origin. However, still, it has demerit due to its non-reproducibility. It once again proved that repeatability of RAPD-PCR is insufficient to distinguish genetic differences among *Salmonella* serovars.

## Authors’ Contributions

SSS carried out the research work; AG planned, designed, and supervised the experiment; BJ assisted in planning designing and execution of work. HK and NK assisted in the collection of sample and laboratory work. All authors read and approved the final manuscript.
